# Supplementary material for: DolphinNext: a distributed data processing platform for high throughput genomics
Source: BMC Genomics. 2020 Apr 19;21:310. doi: 10.1186/s12864-020-6714-x (PMC7168977; doi:10.1186/s12864-020-6714-x)
Supplement: Supplementary file 1 — Additional file 1: Figure S1. RNA-Seq Pipeline. Figure S2. Adapter Removal, Trimmer and Quality Filtering Module. Figure S3. Sequential Mapping Module. Figure S4. BAM Analysis Module. Figure S5. ATAC-Seq pipeline. Figure S6. ChIP-Seq pipeline. [file 12864_2020_6714_MOESM1_ESM.docx]

# **SUPPLEMENTARY FIGURES**

**
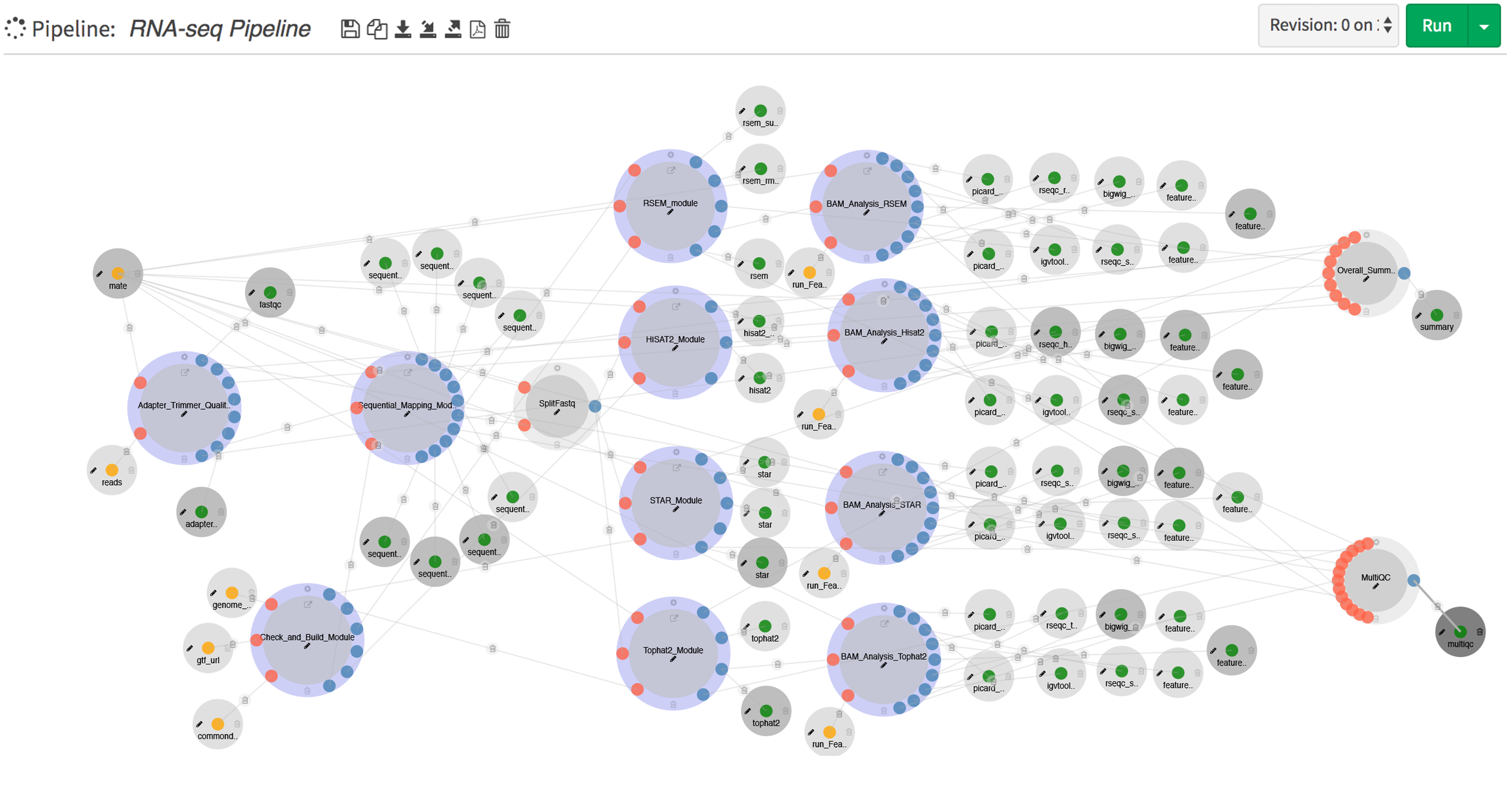
**

**Figure S1.** RNA-Seq Pipeline


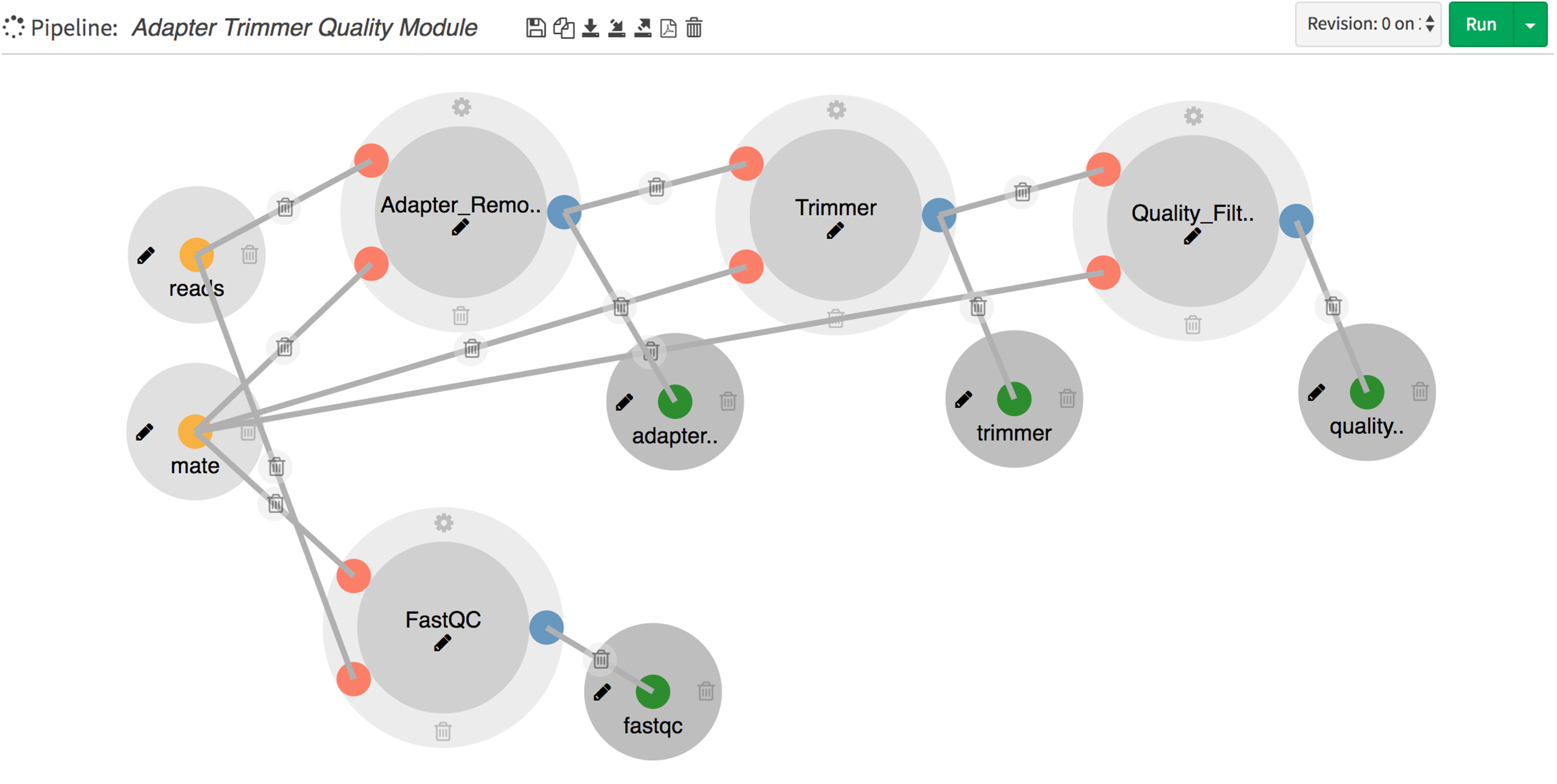


**Figure S2.** Adapter Removal, Trimmer and Quality Filtering Module


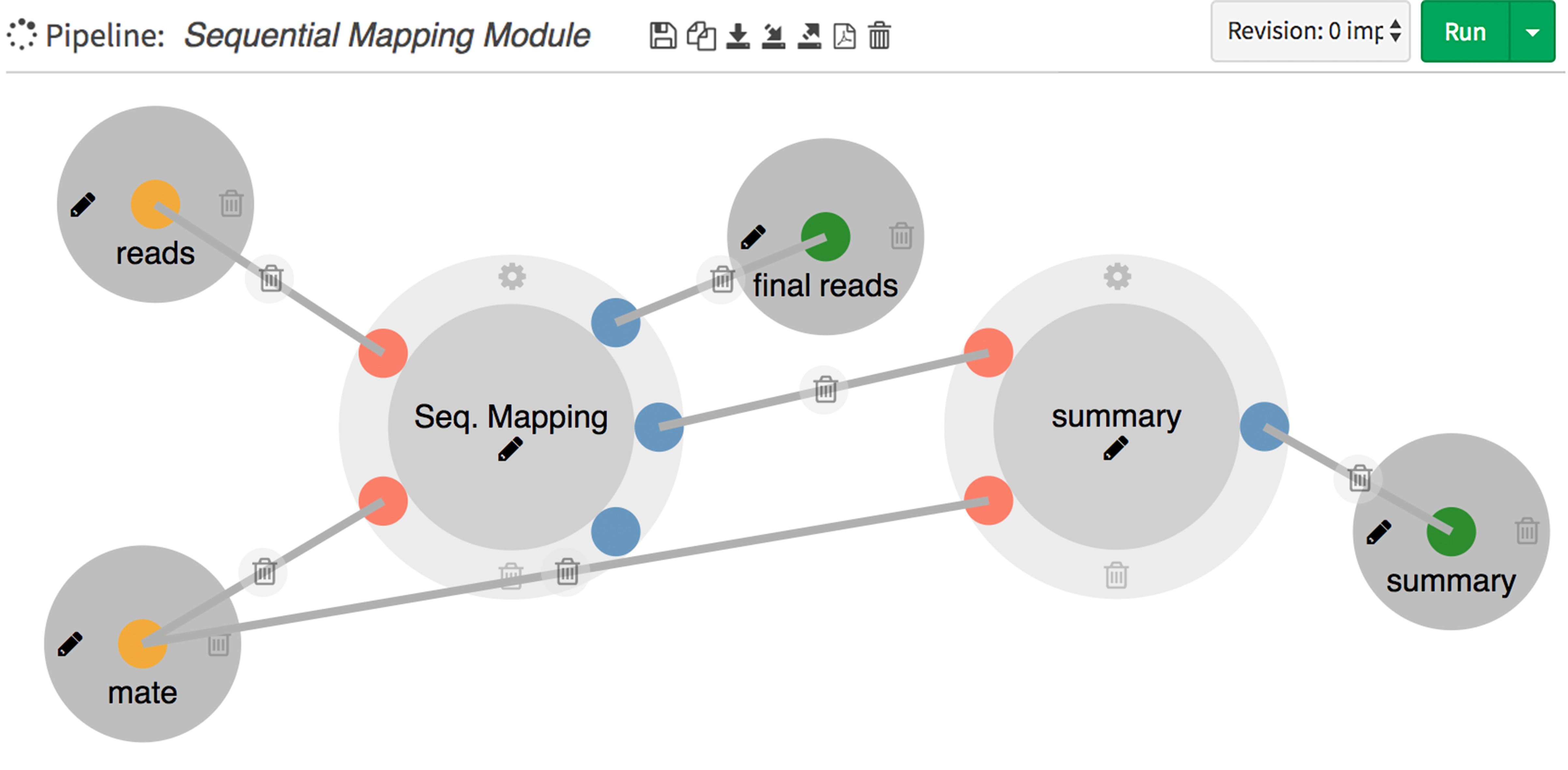


**Figure S3.** Sequential Mapping Module


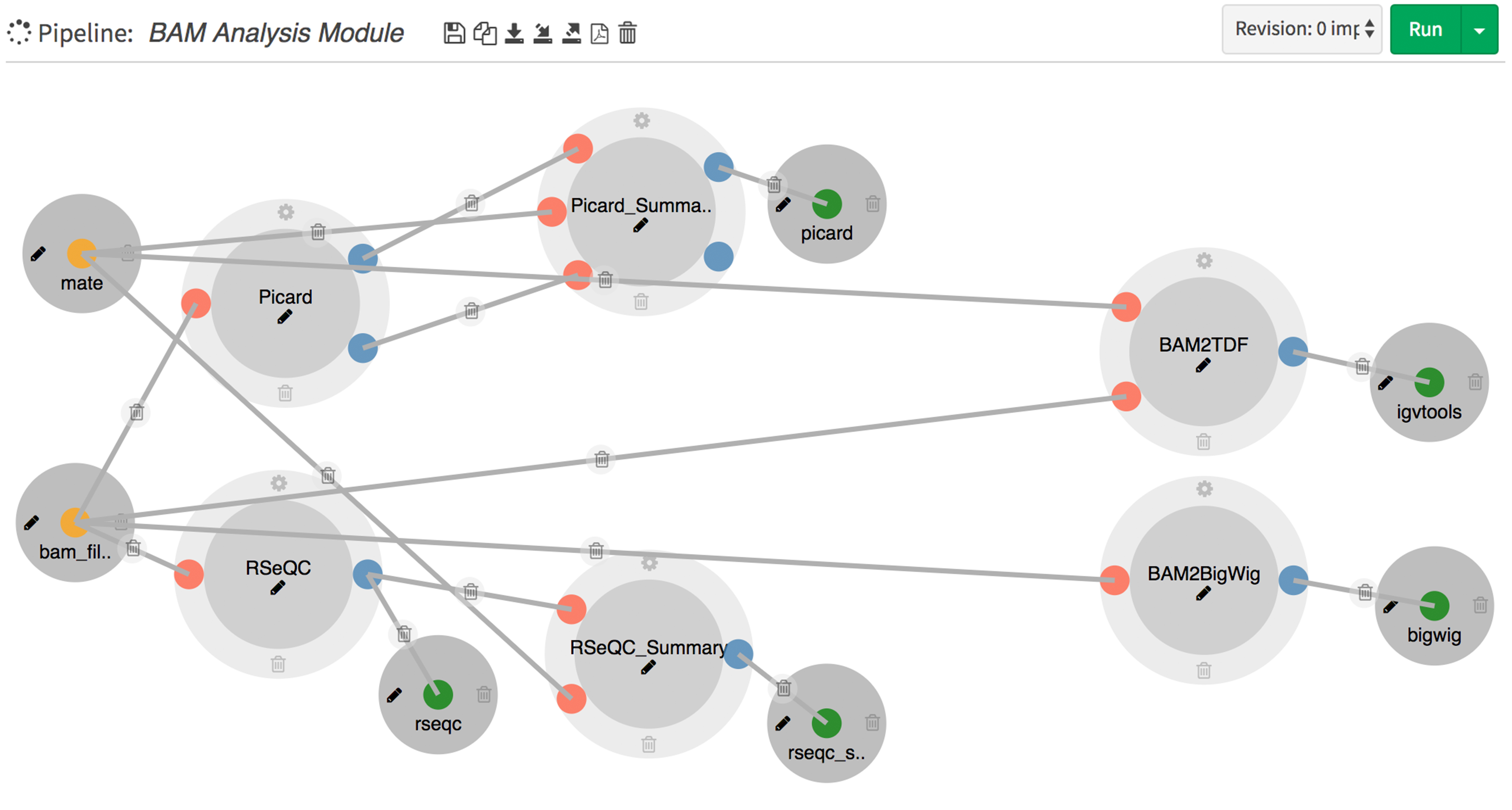


**Figure S4.** BAM Analysis Module


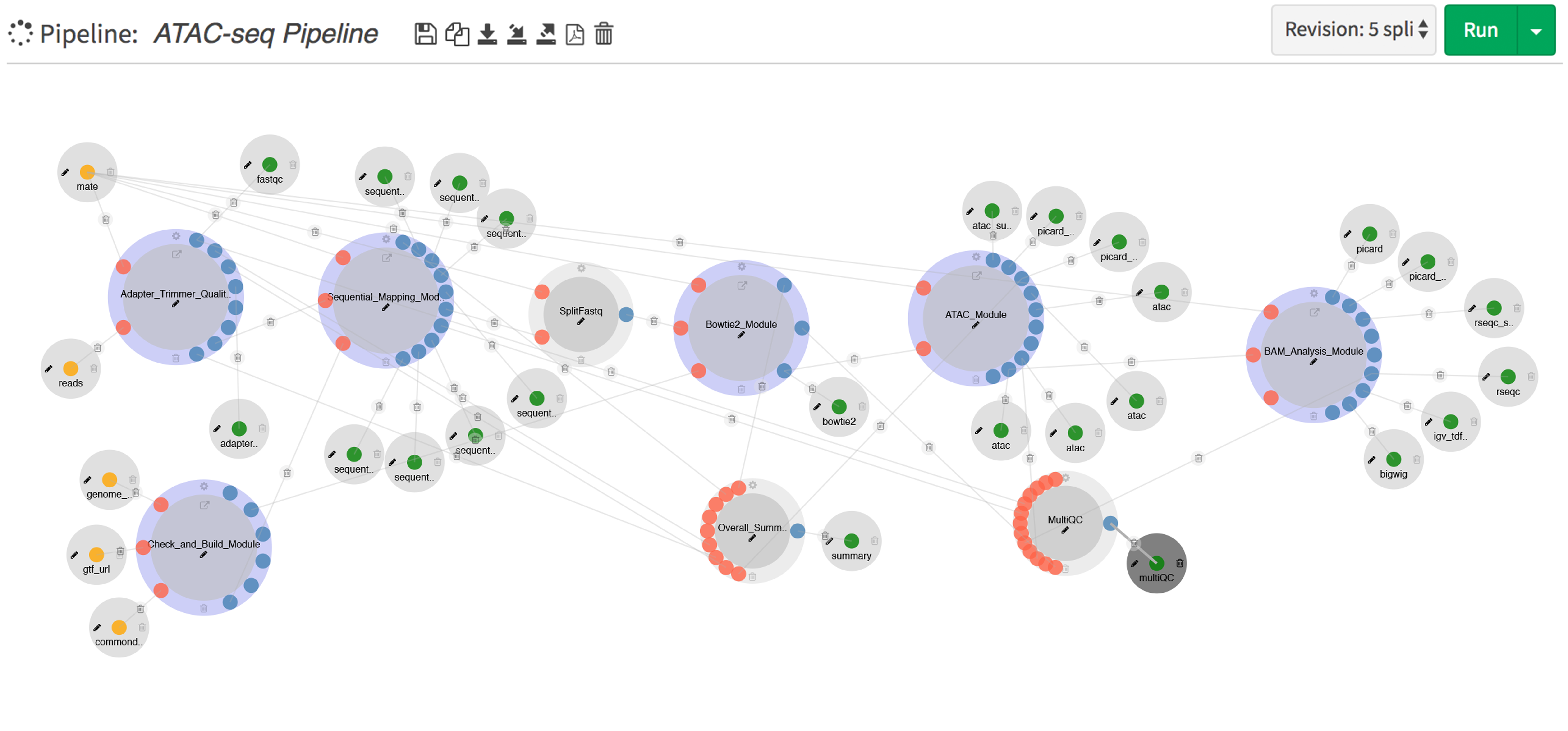


**Figure S5.** ATAC-Seq pipeline


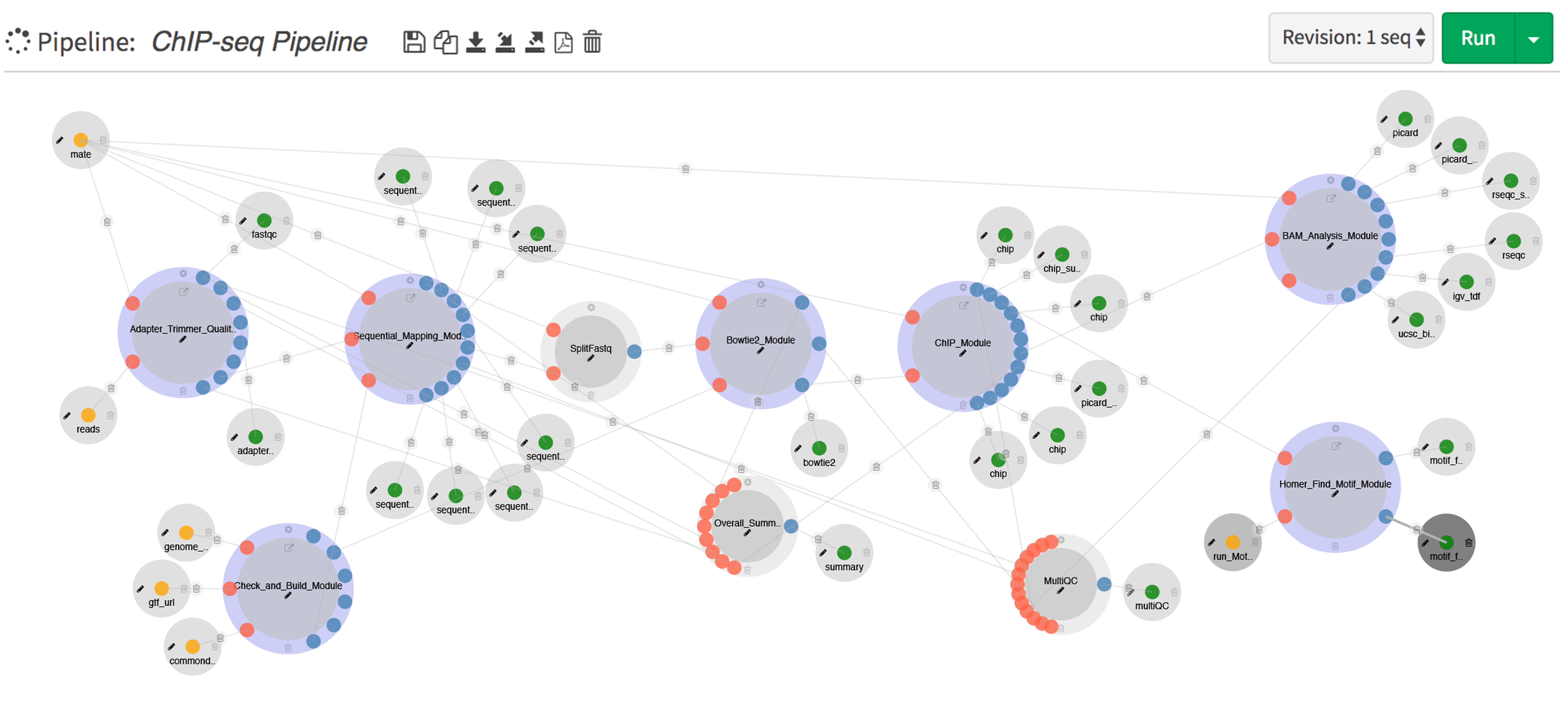


**Figure S6.** ChIP-Seq pipeline
